# An Unusual Combination of Klinefelter Syndrome and Growth Hormone Deficiency in a Prepubertal Child

**DOI:** 10.4274/jcrpe.1225

**Published:** 2014-09-05

**Authors:** Jayanthy Ramesh, Mudiganti Nagasatyavani, Javvadii Venkateswarlu, Jakka Nagender

**Affiliations:** 1 Sai Institute of Endocrinology, Endocrinology, Hyderabad, India; 2 Osmania Medical College and General Hospital, Endocrinology, Hyderabad, India

**Keywords:** short stature, Klinefelter syndrome, Growth hormone deficiency

## Abstract

Klinefelter syndrome (KS) is the most common chromosomal aneuploidy in males. It is very difficult to diagnose this disorder in childhood due to absence of significant manifestations before puberty. These patients usually present with tall stature. We report a case of KS with short stature due to growth hormone deficiency. The boy’s height was below the 3rd centile with significant delay in bone age. He responded well to growth hormone injections. In view of mental subnormality karyotyping was done, which revealed KS (47XXY).

## INTRODUCTION

Klinefelter syndrome (KS) is the most common chromosomal aneuploidy in males. Also, it is the most common genetic cause of azoospermia (1). It is characterized by small testes, tall stature and mental subnormality. We report an unusual case of KS with growth hormone (GH) deficiency and short stature.

## CASE REPORT

**Figure 1:**
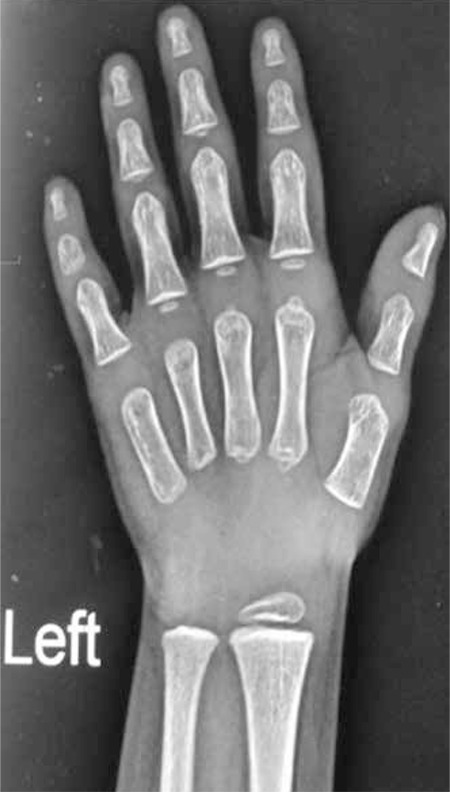
X-ray of the left hand and wrist showing a bone age of less than one year

We report a 3-year-old male child who was referred by his pediatrician for short stature (Figure 1). He was born to parents with no consanguinity, at 36 weeks gestation, by vaginal delivery. His birth weight and length were normal.

**Figure 2:**
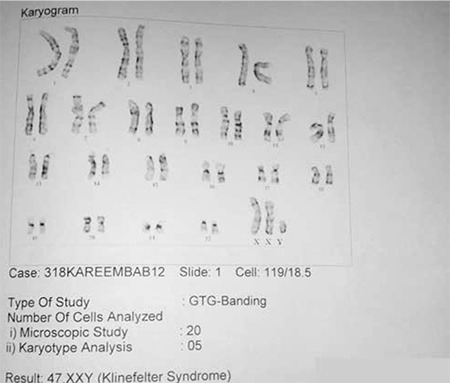
Karyotype showing 47XXY

The boy had delayed motor and language milestones. There was no history of recurrent infections or chronic systemic diseases. His height was 76 cm [height standard deviation score (SDS) -3.7, height age 1 year]. His body proportions were normal for his age. He had no midline facial defects, dysmorphic facies or bony defects. His external genitalia revealed microphallus (stretched penile length of 2 cm, more than 2.5 SD below the mean for the age) with normal external urethral meatus, well-formed scrotum, and palpable prepubertal testes. Other systems were normal. Bone age was delayed by 2 years (Figure 2). The base line investigations and thyroid profile were normal. Serum insulin-like growth factor-1 level was 52.6 ng/mL (age-specific normal range: 49-289 ng/mL) which increased to 132 ng/mL following GH treatment. Clonidine-stimulated GH level was low (0.8 ng/mL) which confirmed GH deficiency. In view of developmental delay and mental subnormality, karyotyping was done and revealed a 47XXY pattern (Figure 2). Chromosomal microarray revealed multiple duplications on X chromosome (of about 55.6mb at cytoband Xp22.33-p11.1, 6.4 mb at cytoband Xq12-q13.1, 27.5mb at cytobandXq13.1-q22.1 and 55.5mb at cytobandXq22.1-q28), implicating presence of an extra copy of X chromosomal complement resulting in KS.

The boy was started on recombinant human GH injections subcutaneously. There were no local or general side effects. After 1 year of treatment, his height increased to 84 cm and height SDS improved from -3.7 to -2.The patient is still receiving recombinant human GH with regular follow-up every 3 months for physical and auxological evaluation.

## DISCUSSION

Tall stature is one of the characteristic features of KS. The clinical phenotype has traditionally been described as tall, with sparse body hair, gynecomastia, small testes and impaired verbal intelligence (2). It is not easy to diagnose KS in childhood because of the absence of significant manifestations before puberty (3). According to a large Danish registry study (4), only 10% of KS men are diagnosed before puberty and only 25% during their lifetime.

The association between KS and GH deficiency has been described in a small number of case reports, but the reason for such an association is not clearly known. Our patient was short due to GH deficiency which was thought to be due to pituitary hypoplasia. Pituitary imaging (MRI) was suggestive of anterior pituitary gland hypoplasia (1.7 mm). Other pituitary functions at this juncture were normal. The association of short stature with delayed bone age, hypoplastic pituitary gland and poor increase in GH level on stimulation tests is suggestive of an isolated GH deficiency. Richer et al (5) reported a few cases of KS with short stature due to presence of an Xq isochromosome. The present case is unique as it is associated with GH deficiency. So far, there are few reported cases of KS and short stature secondary to GH deficiency. Rossodivita et al (6) reported an 8-year-old boy with short stature and cryptorchidism who was later diagnosed to have KS and GH deficiency. Bahillo Curieses et al (7) reported a similar combination in a child receiving GH therapy when evaluated for delayed puberty, but the brain MRI was reported as normal in this patient. Ben-Skowronek et al (8) and Tori et al (9) also reported similar combination of KS and GH deficiency. The diagnosis of KS was made in most of the reported cases when the children failed to enter puberty. Our case is also unique as it was diagnosed in the prepubertal age.

In conclusion, by reporting the findings on this patient, we wish to emphasize the importance of GH testing in pediatric patients with KS, short stature, and delayed bone age.
